# Circulating miRNA-19b as a biomarker of disease progression and treatment response to baricitinib in rheumatoid arthritis patients through miRNA profiling of monocytes

**DOI:** 10.3389/fimmu.2023.980247

**Published:** 2023-03-28

**Authors:** Marzena Ciechomska, Leszek Roszkowski, Tomasz Burakowski, Magdalena Massalska, Anna Felis-Giemza, Adria-Jaume Roura

**Affiliations:** ^1^ Department of Pathophysiology and Immunology, National Institute of Geriatrics, Rheumatology and Rehabilitation, Warsaw, Poland; ^2^ Department of Outpatient Clinics, National Institute of Geriatrics, Rheumatology and Rehabilitation, Warsaw, Poland; ^3^ Biologic Therapy Center, National Institute of Geriatrics, Rheumatology and Rehabilitation, Warsaw, Poland; ^4^ Laboratory of Molecular Neurobiology, Nencki Institute of Experimental Biology, Warsaw, Poland; ^5^ Institute for Research in Biomedicine (IRB Barcelona), The Barcelona Institute of Science and Technology (BIST), Barcelona, Spain

**Keywords:** rheumatoid arthritis, miRNA, baricitinib, monocytes, sequencing, synovial fluids, biomarkers, JAKi inhibitors

## Abstract

**Introduction:**

A number of studies have demonstrated a key role of miRNA isolated from cells, tissue or body fluids as disease-specific biomarkers of autoimmune rheumatic diseases including rheumatoid arthritis (RA) and systemic sclerosis (SSc). Also, the expression level of miRNA is changing during disease development, therefore miRNA can be used as biomarkers monitoring RA progression and treatment response. In this study we have investigated the monocytes-specific miRNA that could serve as potential biomarkers of disease progression observed in sera and synovial fluids (SF) in early (eRA) and advanced (aRA) RA and in RA patients before and 3 months after selective JAK inhibitor (JAKi) -baricitinib treatment.

**Methods:**

Samples from healthy control (HC) (n=37), RA (n=44) and SSc (n=10) patients were used. MiRNA-seq of HC, RA, and SSc monocytes was performed to find versatile miRNA present in different rheumatic diseases. Selected miRNAs were validated in body fluids in eRA (<2 years disease onset) and aRA (>2 years disease onset) and RA patients receiving baricitinib.

**Results:**

Using miRNA-seq, we selected top 6 miRNA out of 95 that were significantly changed in both RA and SSc monocytes compared to HC. To identify circulating miRNA predicting RA progression, these 6 miRNA were measured in eRA and aRA sera and SF. Interestingly, miRNA (-19b-3p, -374a-5p, -3614-5p) were significantly increased in eRA sera vs HC and even further upregulated in SF vs aRA sera. In contrast, miRNA-29c-5p was significantly reduced in eRA sera vs HC and even further decreased in SF vs aRA sera. Kegg pathway analysis predicted that miRNA were involved in inflammatory-mediated pathways. ROC analysis demonstrated that miRNA-19b-3p (AUC=0.85, p=0.04) can be used as biomarker predicting JAKi response.

**Discussion:**

In conclusion, we identified and validated miRNA candidates which were present simultaneously in monocytes, sera, SF and that can be used as biomarkers predicting joint inflammation and monitoring therapy response to JAKi in RA patients.

## Highlights

- Global distribution of miRNA profile is similar in both RA and SSc monocytes
- Selected circulating miRNA can be used as no-invasive biomarkers of joint inflammation in RA patients
- MiRNA-19b can help to predict the responders’ group for baricitinib in RA patients

## Introduction

Autoimmune rheumatic diseases (ARDs) are a group of distinct disorders that share similar clinical, laboratory, and immunological symptoms. Their basic pathobiological finding is: the development of excessive self-reactive and antigen-controlled immune response. Two of the major ARDs are rheumatoid arthritis (RA) and systemic scleroderma (SSc) which, despite the often-different clinical symptoms of the disease, share a similar pathophysiological basis. They can also cause similar complications, including interstitial pneumonia, glomerulonephritis, and serositis (1, 2). Moreover, SSc-related skin involvement and SSc-related arthritis usually respond well to treatment with the drug commonly used to treat RA which is methotrexate (3, 4). This drug belongs to synthetic disease-modifying anti-rheumatic drugs (sDMARD). Monocytes and macrophages play an extremely important role in the pathogenesis and course of both RA and SSc. Indeed, monocytes produce numerous cytokines and chemokines that may be a hallmark of both of these diseases (5, 6). Monocytes are mainly responsible for chronic inflammation and bone erosion in RA, as well as chronic and progressive tissue and organ fibrosis in SSc, which are the main symptoms of these ARDs (7, 8). Thus, modalities produced by monocytes appear to be very good candidates for new markers in both RA and SSc, which can uniquely identify these diseases.

MicroRNA (miRNA) are small (22–24 nt) non-coding RNA sequences that are involved in the negative regulation of gene transcripts and subsequently resulted in their degradation. Therefore, miRNA play an important role in biological processes as post-transcriptional modulators in numerous physiological processes as well as in pathogenesis of ARDs. Indeed, our previous studies revealed that altered expression of specific miRNA (-146b, -26a-3p, -5196, -29b) correlated with clinical parameters including DAS28, mRSS, CRP and ASDAS for RA, SSc, AS (Ankylosing spondylitis). Furthermore, using functional assays we have demonstrated that these miRNA inhibited their target genes including RARA, IFN type I genes, FRA2 and TAB1, respectively in RA and SSc monocytes and SSc fibroblasts (7–10). We have also observed that circulating miRNA-146b was strongly elevated in synovial fluids (SF) of RA patients, suggesting that miRNA-146b can be used as a biomarker of joint inflammation (9). However, a global comparison between miRNA profile of both RA and SSc monocyte population and subsequent validation in sera has not been performed. Thus, the aim of this study was to analyse the global miRNA expression profile of RA and SSc monocytes and to compare their expression pattern to sera-derived RA and SSc miRNA. There is also a great interest in the identification of miRNA that could predict disease progression in order to implement strong therapeutic intervention before joint deformities and functional impairments occur. Indeed, to find a no-invasive biomarker predicting joint inflammation, further qPCR validation of selected miRNA candidates was performed in sera of early and advanced RA and SF. Interestingly, finding unique miRNA monitoring JAK inhibitors (JAKi) therapy response is also of great need to maximize the clinical benefit for the individual RA patient. Relatively modern therapy by JAKi is used as an alternative for biological agents following the failure of the first-line treatment including sDMARD. Therefore, we have measured selected miRNA in RA patients before and 3 months after baricitinib (selective JAK1/2 inhibitor) treatment. Overall, we believe that our study might help clinicians in better patient stratification based on miRNA-driven biomarkers allowing accurate assessments of disease progression and treatment response to JAKi.

## Materials and methods

### Clinical characteristics of the patients

In this study, we used blood from 54 patients who met the 2010 and 2013 American College of Rheumatology/European League Against Rheumatism classification criteria for RA and SSc, respectively (11, 12). Clinical and laboratory parameters of RA (n=44) and SSc (n=10) patients included in the study are displayed in **Table 1**. Healthy controls (HC) (n=37) were characterized as follows: median age was 42 with a range between 22-67 years of age and the ratio of female to male was 29 to 8. This study was approved by the local ethic committee (approval no. KBT-5/3/2019, KBT-6/5/2020) at the National Institute of Geriatrics Rheumatology and Rehabilitation (NIGRiR). The patients’ blood and synovial fluids were obtained from NIGRiR and all patients gave written informed consent prior to inclusion.

**Table 1:** Clinical and laboratory data of RA and SSc patients.

Parameters of RA patients (n = 44)	RA Patients
Age, years, median (range)	52 (23–73)
Sex F/M	38/6
Disease duration, years, median (range)	2,75 (0–30)
Anti-CCP Abs, % (*n*)	79,5% (n=35)
RF, % (*n*)	63,6% (n=28)
CRP, mg/L, median (range)	10 (1-188)
ESR, mm/h, median (range)	24 (3-104)
DAS-ESR, median (range)	5,62 (2,16-8,17)
DAS-CRP, median (range)	5,52 (1,91- 7,78)
**Treatment**	
Methotrexate, % (n)	50% (n=22)
Leflunomide, % (n)	4,5% (n=2)
Sulfasalazine, % (n)	15,9% (n=7)
Hydroxychloroquine, % (n)	13,6% (n=6)
Baricitinib, % (n)	31,8% (n=14)
Methylprednisolone, % (n)	25% (n=11)
Parameters of SSc patients (n = 10)	SSc Patients
Age, years, median (range)	58 (41–86)
Sex F/M	10/0
Disease duration, years, median (range)	4 (0–30)
Modified Rodnan skin score in 17 body surfaces, median (range)	10,5 (4-25)
CRP, mg/L, median (range)	5 (3-12)
**Treatment**	
Cyclophosphamide % (n)	20% (n=2)
Mycophenolate mofetil % (n)	10% (n=1)
Methotrexate, % (n)	10% (n=1)
Prostanoids, % (n)	30% (n = 3)

### Design, participants and cell purification

The blood was collected in EDTA-coated tubes and CD14^+^ monocytes were separated according to the manufacturer’s protocol with the CD14^+^MACS beads isolation kit (Miltenyi-Biotec, The Netherlands) as previously described (9). In experiments measuring cell-free miRNA, we used sera from SSc patients, sera from early RA (eRA) with <2 years disease onset, and advanced RA (aRA) with >2 years disease onset and plasma from RA patients receiving baricitinib before and 3 months after therapy.

### Global miRNA profiling of RA and SSc monocytes

MiRNA from monocytes was isolated using a miRNeasy kit (Qiagen, UK) according to the manufacturer’s protocol. Global miRNA profiling was performed in 10 HC, 10 SSc and 12 RA monocytes. The results from miRNA-seq were normalized by the mirDeep2 pipeline and subsequently analysed using R programming language. The processed fasta file was mapped to the human genome using the mirAligner software (version 3.5). Following post-processing analysis, miRNA with adjusted p-values < 0.05 and false discovery rate (FDR) < 0.05 were considered significantly differently expressed between the HC and RA/SSc group. In order to increase the power and integrated information of the miRNA-seq analysis, we used miRNet webtool and the Reactome database to identify and highlight the target genes related to inflammatory and immune-related pathways, as well as investigated the miRNA-gene network. We have focused only on pathways that were significantly enriched based on corrected p-values < 0.05 from the whole list of miRNA.

### Circulating miRNA validation in RA and SSc sera and RA synovial fluids

The volume of 300 µl of sera or plasma from SSc (n=10), RA (n=44) patients and and HC (n=37) patients was used to isolate circulating miRNA using NucleoSpin^®^ miRNA plasma/serum (Macherey–Nagel, Germany) according to the manufacturer’s protocol. Circulating miRNA expression level was validated using TaqMan ^®^ microRNA RT Kit (Thermo Fisher Scientific, USA) and TaqMan^®^ MicroRNA Assays. The IDs of the probes are: hsa-miR-589-3q (479072_mir), hsa-3614-5p (478836_mir), hsa-miR-374a-5p (478238_mir), hsa-miR-29c-3p (479229_mir), hsa-miR-19b-3p (478264_mir), hsa-miR-503-5p (478143_mir), has-miR-16-5p (477860_mir), all from ThermoFisher Scientific (USA). The expression levels of circulating miRNA in SSc and RA patients were relative to the average HC (arbitrarily set at 1) and were calculated using the following equation: 2^(-delta delta CT). All samples were normalized to miRNA-16-5p as an internal control.

### KEGG enrichment analysis

KEGG pathways enrichment analysis of selected 6 miRNA was performed using DIANA-mirPath software v.3. This algorithm was used to predict the interaction between 6 miRNA and their mRNA targets based on the coding sequence (CDS). Illustrated interactions were previously filtered by FDR correction, p-value threshold less than 0.05, and Fisher’s Exact Test.

### Statistical analysis

To determine the statistical significances, we performed Mann-Whitney test for unpaired samples and Wilcoxon matched-pairs signed-rank test for non-normally distributed data. Error bars were defined as S.E.M. P-values were expressed as follows: ns for not significant; 0.05 > P > 0.01 as *; 0.01 > P > 0.001 as **; P < 0.001 as ***, P <0.0001 as ****. To validate accuracy of miRNA-derived biomarkers, receiver-operating characteristics (ROC) curve was performed. The cut-off value was established based on maximal Youden index. GraphPad Prism version 4.03 software was used.

## Results

### Identification of miRNA that were common in both RA and SSc monocytes

**Figure 1:**
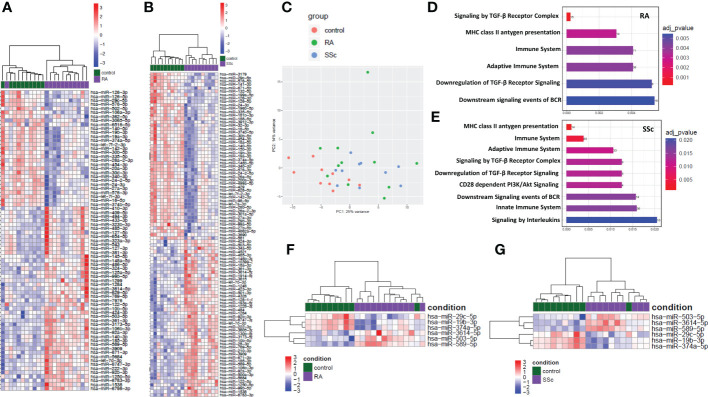
Global analysis and distribution of miRNA-seq in RA and SSc monocytes. Heatmap of significantly dysregulated miRNA in RA **(A)** and SSc **(B)** monocytes compared to HC. Principal component analysis (PCA) plot of global miRNA distribution in RA (green), SSc (blue) and HC (red) monocytes **(C)**. Enrichment analysis of abundantly expressed miRNA regulating selected genes involved in immune pathways in RA **(D)** and SSc monocytes **(E)**. Heatmap analysis of selected miRNA in RA **(F)** and SSc **(G)** monocytes compared to HC.

In order to find common miRNA for both RA and SSc in monocytes compared to HC we have performed global miRNA profiling using miRNA-seq. The heatmap analysis demonstrated top 75 for RA (**Figure 1A**) and 95 for SSc (**Figure 1B**) significantly down-regulated and up-regulated miRNA that were changed in these diseases compared to HC. The principal component analysis (PCA) plot demonstrated that the global miRNA expression pattern was distributed similarly between RA and SSc patients, but was separated from HC, suggesting similarities and overlap in miRNA clustering between RA and SSc monocytes (**Figure 1C**). In addition, Reactome database revealed that miRNA were predicted to regulate similar immune pathways in both RA and SSc monocytes including innate and adaptive immune system regulation, TGF-β signaling, and MHC class II antigen presentation among others (**Figures 1D**, **E**). Subsequently, we selected 6 miRNA which had the same expression pattern both in RA and SSc monocytes compared to HC (**Figures 1F, G**). Indeed, heatmap analysis revealed that 3 miRNA (-19b-3p, -29c-5p, -374a-5p) were downregulated, whereas 3 other miRNA (-503-5p, -589-5p, -3614-5p) were upregulated in both RA and SSc monocytes. We also investigated genes and their signaling pathways that can be directly targeted by selected 6 miRNA. Using the miRNet web tool, we identified and highlighted genes associated with inflammatory and immune-related pathways (**Supplementary Figure S1**). We assessed only significantly enriched miRNA-gene networks based on corrected p-values < 0.05.

### Validation of selected miRNA in RA and SSc sera

**Figure 2:**
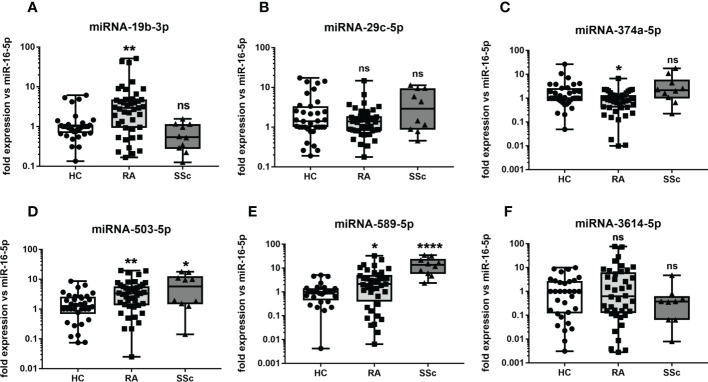
The level of circulating miRNA in RA and SSc patients. The level of miRNA-19b-3p **(A)**, miRNA-29c-5p **(B)**, miRNA-374a-5p **(C)**, miRNA-503-5p **(D)**, miRNA-589-5p **(E)**, miRNA-3614-5p **(F)** in sera of RA and SSc patients compared to HC. P-values were expressed as follows: ns for not significant; 0.05 > P > 0.01 as *; 0.01 > P > 0.001 as **; P < 0.001 as ***, P <0.0001 as ****.

We then measured the expression of selected miRNA in RA and SSc sera in order to compare the cell-specific miRNA expression to cell-free miRNA in both ARDs. Samples used to measure cell-specific miRNA and cell-free miRNA expression were isolated from the same SSc patients. In HC and RA cell-free miRNA analysis, we used a larger cohort than the primary cell-specific miRNA used for miRNA-seq. In **Figures 2B-F** it can be seen that expression of selected miRNA (-29c-5p, -374a-5p, -503-5p, -589-5p, -3614-5p) in RA sera had similar expression pattern as seen in monocytes. In contrary, miRNA-19b-3p was significantly (p= 0.0016) increased in RA sera (**Figure 2A**) and downregulated in RA monocytes from the miRNA-seq analysis (**Figure 1F**). In SSc sera, the expression of miRNA-19b-3p and miRNA-589-5p had the same expression pattern as seen in SSc monocytes. The rest of circulating SSc miRNA candidates (miRNA-29c-5p, -374a-5p, -503-5p, -3614-5p) did not differ significantly compared to HC. Overall, these data suggest that 3 cell-free miRNA candidates in RA sera had the same expression pattern as seen in RA monocytes. While in SSc sera only 2 cell-free miRNA had the same expression pattern as seen in SSc monocytes, probably due to the limited sample size of the SSc group. Therefore, we have then performed a deeper analysis only on RA patients in order to find circulating miRNA as biomarkers of disease progression and treatment response.

### The expression of circulating miRNA candidates in sera and synovial fluids of RA patients

**Figure 3:**
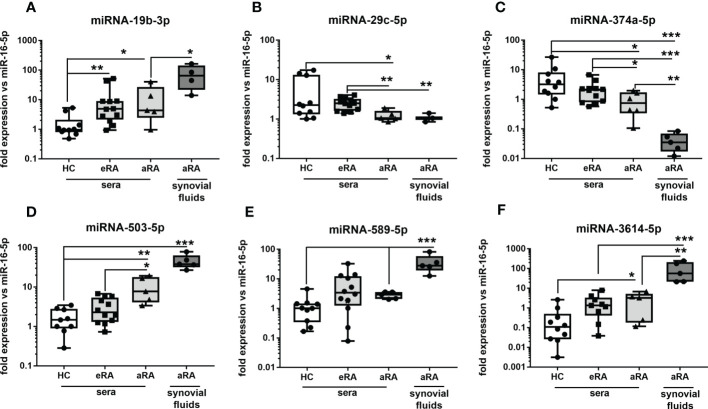
The levels of circulating miRNA in body fluids of eRA and aRA patients. The levels of miRNA-19b-3p **(A)**, miRNA-29c-5p **(B)**, miRNA-374a-5p **(C)**, miRNA-503-5p **(D)**, miRNA-589-5p **(E)**, miRNA-3614-5p **(F)** in sera or eRA and aRA and SF of aRA patients compared to HC. P-values were expressed as follows: ns for not significant; 0.05 > P > 0.01 as *; 0.01 > P > 0.001 as **; P < 0.001 as ***, P <0.0001 as ****.

The next step was to measure selected miRNA in sera of eRA and aRA, but also in SF of aRA. This stage allowed us to identify unique miRNA predicting joint inflammation and ultimately monitoring RA progression based on previously selected cell-specific miRNA. In **Figures 3A, D–F**, it can be seen that the expression of miRNA (-19b-3p, -503-5p, -589-5p, -3614-5p) were gradually enhanced in eRA sera through high abundance in aRA sera, and the highest expression levels were seen in SF of aRA. On the other hand, we have observed a gradual reduction of miRNA-29c-5p and miRNA-374a-5p in eRA sera through low abundance in aRA sera and the lowest expression levels were seen in SF of aRA (**Figures 3B, C**). Overall, these data suggest that cell-free circulating miRNA (-19b-3p, -503-5p, -589-5p, -3614-5p, 29c-5p, -374a-5p) can be used as biomarkers predicting joint inflammation and subsequently cartilage destruction in early-stage of RA.

### Functional enrichment analysis of selected miRNA

**Figure 4:**
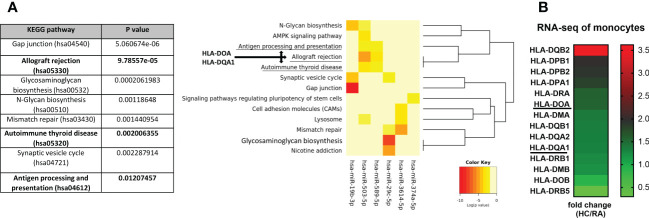
KEGG pathway analysis of selected 6 miRNA candidates and their potential target genes. The identification of specific pathways **(A)** and their downstream genes **(B)** including HLA-DOA and HLA-DQA1, which were predicted to be negatively regulated by significantly changed miRNA in RA patients, using the Diana-miPath tool. The fold change analysis of expression level of HLA class II genes between HC and RA monocytes from RNA-seq **(B)** based of FDR < 0.01 and p-values < 0.05.

To further investigate the function of selected 6 miRNA candidates, KEGG pathway analysis was conducted using the online DIANA-mirPath database. Interestingly, 3 out of the top 8 pathways of the enrichment analysis were predicted to be associated with autoimmunity including allograft rejection (hsa05330), autoimmune thyroid disease (hsa05320), and antigen processing and presentation (hsa04612), through significant negative regulation of HLA-DOA and HLA-DQA1 genes by all selected miRNA (**Figure 4A**). This analysis clearly indicates that selected miRNA candidates were involved in autoimmunity. In addition, RNA-seq analysis confirmed reduced expression of 12 (including HLA-DOA - 1.5 fold reduction and HLA-DQA1 – 1.3 fold reduction) out of 14 detected HLA class II genes in RA monocytes compared to HC (**Figure 4B**). These results suggest that selected miRNA candidates may negatively regulate HLA class II in RA monocytes. In **Supplementary Table S3** and **Supplementary Figure S4**, there are more detailed analyses of the miRNA-gene network to illustrate the effects of miRNA deregulation in RA and SSc monocytes.

### The expression of circulating miRNA candidates in RA patients before and after JAKi therapy

**Figure 5:**
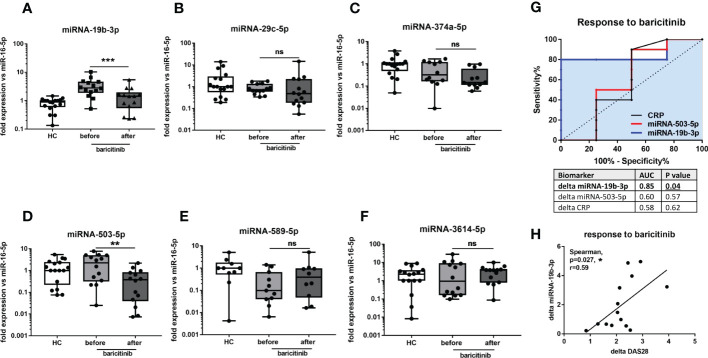
The levels of circulating miRNA in HC and RA patients upon baricitinib therapy. The levels of miRNA-19b-3p **(A)**, miRNA-29c-5p **(B)**, miRNA-374a-5p **(C)**, miRNA-503-5p **(D)**, miRNA-589-5p **(E)**, miRNA-3614-5p **(F)** before and 3 months after baricitinib treatment in RA patients compared to HC. ROC analysis of changes/delta in miRNA-19b-3p and miRNA-503-5p expression and CRP level upon JAKi therapy **(G)**. Correlation analysis between the expression of delta miRNA-19b-3p and delta DAS28 in baricitinib-treated RA patients **(H)**. P-values were expressed as follows: ns for not significant; 0.05 > P > 0.01 as *; 0.01 > P > 0.001 as **; P < 0.001 as ***, P <0.0001 as ****.

We have also measured selected miRNA in RA patients before and 3 months after JAKi therapy in order to test if these 6 miRNA can be useful as biomarkers for monitoring treatment response. In **Figure 5A** it can be seen that circulating miRNA-19-3p was significantly (p= 0.0001) reduced in all RA patients after baricitinib treatment reaching a similar level as HC. Also, miRNA-503-5p was significantly (p= 0.0085) downregulated following JAKi therapy in 12 out of 14 RA patients (**Figure 5D** and **Supplementary Figure S2**). Simultaneously, the average DAS28 score was significantly reduced (p= 0.0001) from 5.8 to 3.3 in all RA patients upon baricitinib administration (**Supplementary Figure S2**). The rest miRNA (-29c-5p, -374a-5p, -589-5p, -3614-5p) did not reach statistical significance before and after baricitinib treatment (**Figure 5B, C, E, F**). Finally, to evaluate the diagnostic potential of miRNA-19b-3p and miRNA-503-5p as biomarkers predicting JAKi treatment response compared to CRP, ROC analysis was introduced. In **Figure 5G**, it can be seen that changes/delta in miRNA-19b-3p expression (AUC=0.85, p=0.04) before and after drug treatment were more specific to identify the responder group (reduction in DAS28 of more than 2) than changes in miRNA-503-5p (AUC=0.6, p=0.57) or changes in CRP level (AUC=0.58, p=0.62). In addition, it has been demonstrated significant (p=0.027) positive correlation between the expression of delta miRNA-19b-3p and delta DAS28 in baricitinib-treated RA patients (**Figure 5H**). Overall, these data indicate that miRNA-19b-3p can be used as a biomarker for monitoring both disease progression and JAKi treatment response represented by strong DAS28 reduction in RA patients.

## Discussion

In this paper, we have identified unique miRNA candidates expressed in monocytes that were simultaneously present in both ARDs (RA and SSc) using global miRNA-seq. Further qPCR validation of selected miRNA candidates in body fluids of eRA and aRA and RA patients following 3 months JAKi therapy has allowed us to decipher circulating miRNA as universal diagnostic biomarkers of disease progression and JAKi treatment response.

Several studies have demonstrated a key role of miRNA isolated from cells, tissue, or body fluids as disease-specific biomarkers of ARDs including RA and SSc (13–15). In the previous study, we demonstrated that sera-derived miRNA-5196 was increased in SSc, RA and AS patients compared to HC (16). On the other hand, the expression level of miRNA was changing during disease development or therapeutic intervention. Indeed, we have shown that the level of miRNA-5196 was reduced in 80% of RA patients and all AS patients responding to anti-TNF-α therapy (16). In addition, miRNA-5196 was a better biomarker for monitoring TNFi-response than CRP level. The same miRNA-5196 was also investigated in SSc monocytes and was involved in the reduction of profibrotic properties of SSc monocytes (10). In contrast, miRNA-146b was elevated in cell-free fraction (sera and SF) but was reduced in RA monocytes (9). A few other studies also examined the expression of the same miRNA in PBMCs, body fluids, or different tissues. For instance, 49 miRNA candidates were found to be altered in both PBMCs (out of 91) and plasma-derived extracellular vesicles (out of 150) isolated from patients with Chronic Fatigue Syndrome compared to HC. Interestingly, one of the most differentially expressed miRNA in this study published by Almenar-Perez et al., was miRNA-374a (17). Also, the level of miRNA-374a was significantly increased in SF compared to sera isolated from osteoarthritis (OA) patients (18). Similarly, the level of miRNA-29 was significantly increased in both PBMCs and SF of OA patients and correlated positively with age, body mass index (BMI) and Kellgren-Lawrence X-ray classification (19). These miRNA (-374a-5p and -29c-5p) were also selected as candidates in our study (**Figures 1F**, **2B, C**). The other study found that 143 miRNA were present in 61 different tissue biopsies of different organs (20). On the other hand, the results from prostate cancer study demonstrated revers expression of miRNAs (-125b, -126, -141, -143, -221) in tumour tissue compared to plasma (21). Also, expression patter of miRNA-21 and let-7a was opposite between sera and breast cancer tissue (22). Overall, these data suggest that miRNA expression is unique and can be either up- or downregulated across various tissues, cells, or body fluids, which is in line with our results demonstrating that selected monocytes-specific miRNA can be also present in cell-free fractions with similar (miRNA-503-5p, -589-5p, -374a-5p) or opposite expression pattern between sera and monocytes (miRNA-19b-3p).

Circulating miRNA have emerged as minimally-invasive biomarkers that are associated with disease progression and treatment response, thus we have also measured miRNA in RA body fluids to find unique biomarkers that would predict joint inflammation and subsequently joint damage at the early stage. In our paper we have measured selected miRNA. It is known that circulating miRNAs originate as a by-product of dead cells or exosomes (23). Therefore, we decided to measure miRNA in monocytes because these cells are the second population after lymphocytes consisting of even 30 percent of PBMCs. Also macrophages, which differentiate from monocytes, play a role in clearance by phagocytosis which is linked to apoptosis and necrosis (24). We believe this approach allowed us to select versatile miRNA candidate present in both monocyte-derived fractions and in sera-derive fractions in order to increase the power and importance of potential biomarkers. Indeed, based on miRNA-seq of RA and SSc monocytes we have selected 6 candidates, which were present in both diseases. These miRNA candidates (-19b-3p, -29c-5p, -374a-5p, -503-5p, -589-5p, -3614-5p) were subsequently validated in sera of patients with eRA and compared to sera and SF of patients with aRA. This experiment allowed us to test whether selected miRNA candidates present in sera from aRA may reflect the situation observed in the inflamed joint cavity or even predict these changes by measuring miRNA in sera from eRA. In our study, we have demonstrated that expression of miRNA-19b-3p and miRNA-3614-5p was gradually enhanced in eRA sera through high abundance in aRA sera, and the highest expression level was seen in synovial fluid of aRA. In other studies, it has been indicated that an increased plasma level of miRNA-19b was a good predictor of the risk of OA development and was positively correlated with disease severity (25). On the other hand, TLR2-mediated activation of synovial fibroblasts from RA patients downregulated the level of miRNA-19a/b (26). Whereas, overexpression of miRNA-19a/b reduced MMP-3 and IL-6 secretion. Overall, our and other data suggest that miRNA-19 could be used as a promising biomarker for diagnosis and disease severity of knee inflammation in RA and OA. We have also observed a gradual reduction of miRNA-29c-5p and miRNA-374a-5p in eRA sera through low abundance in aRA sera and the lowest expression level was seen in SF of aRA. A similar pattern of down-regulated expression of the miRNA-29 family was seen in the thymus of the autoimmune disease Myasthenia gravis compared to HC (27). Conversely, miRNA-29c was significantly increased in late-stage OA SF compared to early-stage OA SF (28). This discrepancy in miRNA-29c expression in SF to our finding could be explained by more inflammatory-driven joint damage in RA compared to more mechanical wear and tear on joints observed in OA. We have also revealed that miRNA-503-5p was upregulated in RA and SSc, but did not reach statistical significance in SSc. This observation is in line with the previously published data demonstrating enhanced secretion of miRNA-503 in either extracellular vesicles (EVs), secreted by inflammatory macrophages, or the EVs from peripheral blood mononuclear cells of atherosclerosis patients (29). In addition, miRNA-503 expression was upregulated in SSc skin tissue and promotes angiotensin-II induced cardiac fibrosis (30, 31). We have also measured the level of miRNA candidates before and after baricitinib treatment, in order to identify potentially unique miRNA that are changing during JAKi therapy. Such biomarkers could find broad implications for monitoring response to drug treatment. Interestingly, we noticed that only miRNA-19b-3p and miRNA-503-5p were significantly reduced upon JAKi treatment. However, following ROC analysis only miRNA-19b-3p had a strong potential to be a biomarker predicting reduced disease activity upon 3 months baricitinib treatment. Previously only one study demonstrated that the levels of miRNA-432-5p and miRNA-194-5p were downregulated (out of 61 miRNA candidates) in RA patients achieving clinical remission upon another JAKi - tofacitinib treatment (32). This suggests that only selected miRNA are changing during JAKi therapy. Certainly, the limitation of our study is the number of RA patients before and after baricitinib treatment, which should be justified on larger cohort in order to truly prove that miRNA-19b-3p can be used as biomarker monitoring tsDMARDs-response.

In conclusion, using a global approach, we have selected unique miRNA profiles (miRNA-19b-3p, miRNA-29c-3p, miRNA-374a-5p, miRNA-503-5p, miRNA-589-3q, miRNA-3614-5p) observed not only in monocytes from RA and SSc but also present in sera and SF in RA patients as no-invasive biomarkers of joint inflammation that are predicted to negatively regulate selected HLA class II genes. Further miRNA validation, allowed us, to identify the additional function of miRNA-19b-3p as a new biomarker of disease activity that predicts treatment response to baricitinib in RA patients.

## Data availability statement

The datasets presented in this study are deposited in the European Genome-phenome Archive (EGA) repository (EGA-box-1726). However, as the datasets contain sensitive information (DNA and RNA sequences from humans) access to this data is limited/controlled, further inquiries can be directed to the corresponding author/s.

## Ethics statement

The studies involving human participants were reviewed and approved by KBT-5/3/2019, KBT-6/5/2020. The patients/participants provided their written informed consent to participate in this study.

## Author contributions

All authors were involved in drafting the article or revising it critically for important intellectual content, and all authors approved the final version to be published. MC had full access to all of the data in the study and takes responsibility for the integrity of the data and the accuracy of the data analysis. Study conception and design, integrity of the paper, project management: MC. Manuscript writing: MC, A-JR, LR. Acquisition of data: MC, TB, AF-G, MM, LR. Analysis and interpretation of data: MC, A-JR.

## Supplementary material

Supplementary Figure 1 Enrichment analysis of 6 selected miRNA and predicted their target genes which are involved in immune pathways dysfunction both in RA and SSc monocytes.

Supplementary Figure 2 The levels of circulating miRNA and CRP in RA patients upon baricitinib therapy. The levels of miRNA-19b-3p **(A)**, miRNA-503-5p **(B)**, CRP **(C)** and DAS28 **(D)** before and 3 months after baricitinib treatment in RA patients.

Supplementary Table 3 The list of particular miRNAs (miRNA-19b-3p, miRNA-29c-3p, miRNA-374a-5p, miRNA-503-5p, miRNA-589-3q, miRNA-3614-5p) and the genes that have been dysregulated in RA and SSc.

Supplementary Figure 4 MiRNA-gene network analysis illustrating the effects of miRNA deregulation in RA and SSc.
