# Supplementary figures and images for: Circulating miRNA-19b as a biomarker of disease progression and treatment response to baricitinib in rheumatoid arthritis patients through miRNA profiling of monocytes
Source: Front Immunol. 2023 Mar 28;14:980247. doi: 10.3389/fimmu.2023.980247 (PMC10086423; doi:10.3389/fimmu.2023.980247)

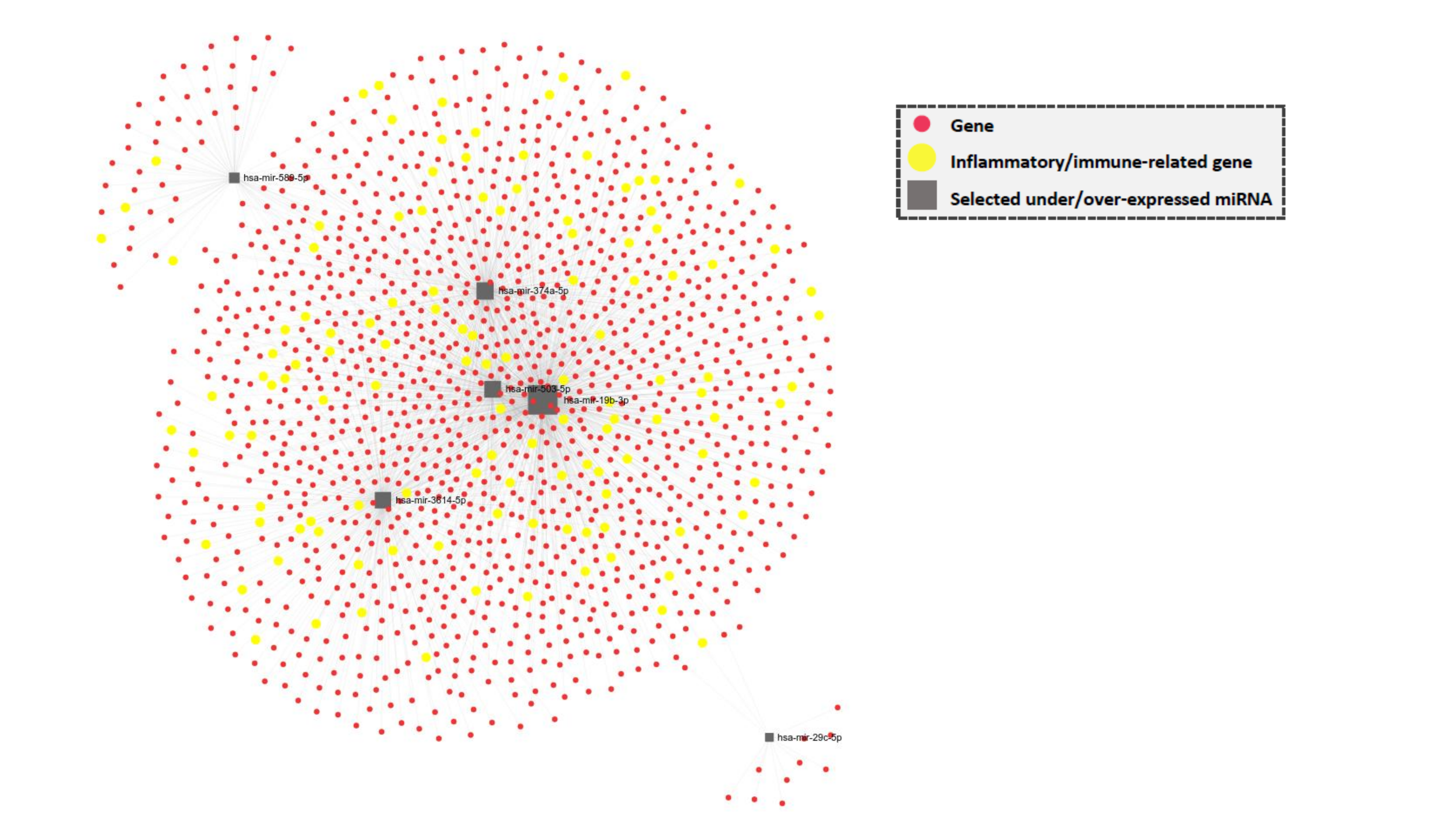

Supplement: Supplementary Figure 1 — Enrichment analysis of 6 selected miRNA and predicted their target genes which are involved in immune pathways dysfunction both in RA and SSc monocytes. [file Image_1.tif]

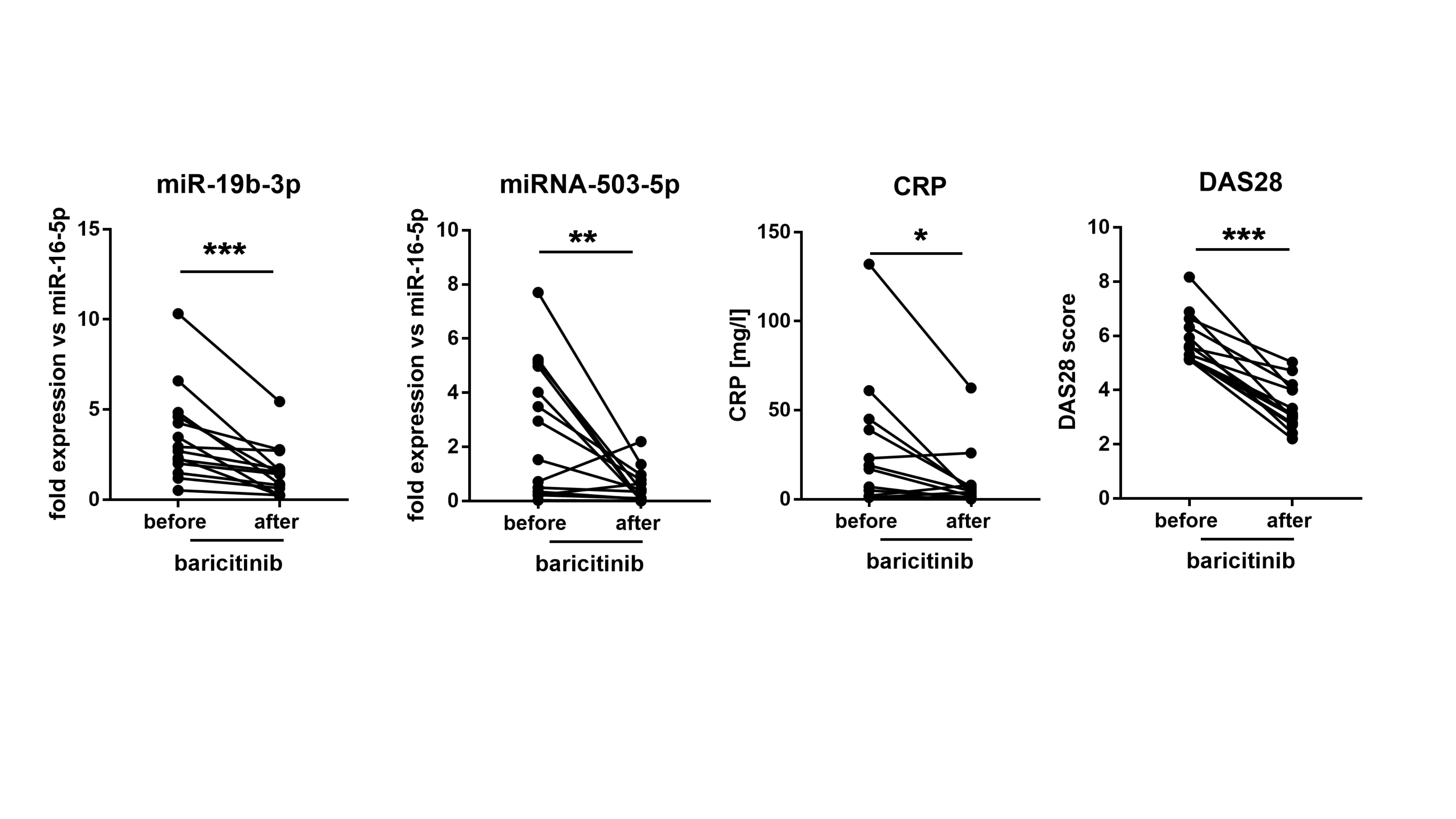

Supplement: Supplementary Figure 2 — The levels of circulating miRNA and CRP in RA patients upon baricitinib therapy. The levels of miRNA-19b-3p (A), miRNA-503-5p (B), CRP (C) and DAS28 (D) before and 3 months after baricitinib treatment in RA patients. [file Image_2.tif]
