# Supplementary material for: Circulating miRNA-19b as a biomarker of disease progression and treatment response to baricitinib in rheumatoid arthritis patients through miRNA profiling of monocytes
Source: Front Immunol. 2023 Mar 28;14:980247. doi: 10.3389/fimmu.2023.980247 (PMC10086423; doi:10.3389/fimmu.2023.980247)
Supplement: Supplementary Figure 4 — MiRNA-gene network analysis illustrating the effects of miRNA deregulation in RA and SSc. [file DataSheet_1.pdf]

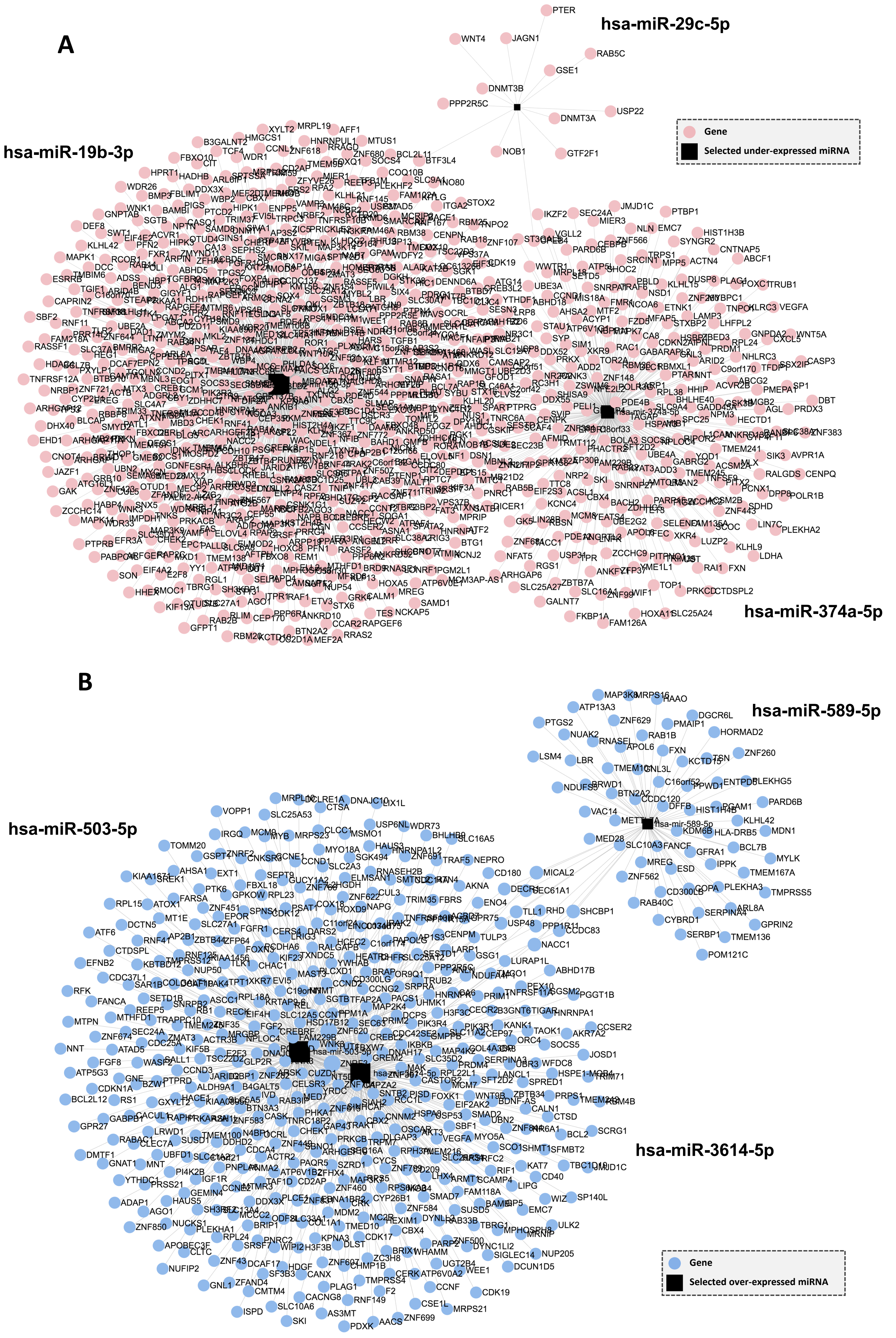
A
hsa-miR-29c-5p
hsa-miR-19b-3p
hsa-miR-374a-5p
Gene
Selected under-expressed miRNA
B
hsa-miR-589-5p
hsa-miR-503-5p
hsa-miR-3614-5p
Gene
Selected over-expressed miRNA
